# The effect of larval nutritional deprivation on the life history and DDT resistance phenotype in laboratory strains of the malaria vector *Anopheles arabiensis*

**DOI:** 10.1186/1475-2875-12-44

**Published:** 2013-02-01

**Authors:** Shüné V Oliver, Basil D Brooke

**Affiliations:** 1 Vector Control Reference Laboratory, Centre for Opportunistic, Tropical and Hospital Infections, National Institute for Communicable Disease, 1 Modderfontein Road, Sandringham, Johannesburg, South Africa; 2 Malaria Entomology Research Unit, School of Pathology, Faculty of Health Sciences, University of the Witwatersrand, 7 York Road, Parktown, Johannesburg, South Africa

**Keywords:** *Anopheles arabiensis*, Larval nutrition, DDT resistance, Adult body size

## Abstract

**Background:**

*Anopheles arabiensis* is a major malaria vector in Africa. It thrives in agricultural areas and has been associated with increased malaria incidence in areas under rice and maize cultivation. This effect may be due to increased adult size and abundance as a consequence of optimal larval nutrition. The aim of this study was to examine the effect of larval nutrition on the life history and expression of insecticide resistance in adults of laboratory reared *An*. *arabiensis*.

**Methods:**

Larvae drawn from an insecticide susceptible *An*. *arabiensis* strain (SENN) as well as a DDT-resistant strain (SENN-DDT) were subjected to three fasting regimes: 1 mg of food per larva offered once per day, once every second day and once every third day. Control cohorts included larvae offered 1 mg food thrice per day. The rate of larval development was compared between matched cohorts from each strain as well as between fasted larvae and their respective controls. The expression of DDT resistance/tolerance in adults was compared between the starved cohorts and their controls by strain. Factors potentially affecting variation in DDT resistance/tolerance were examined including: adult body size (wing length), knock-down resistance (*kdr*) status and levels of detoxification enzyme activity.

**Results and conclusion:**

*Anopheles arabiensis* larval development is prolonged by nutrient deprivation and adults that eclose from starved larvae are smaller and less tolerant to DDT intoxication. This effect on DDT tolerance in adults is also associated with reduced detoxification enzyme activity. Conversely, well fed larvae develop comparatively quickly into large, more DDT tolerant (SENN) or resistant (SENN-DDT) adults. This is important in those instances where cereal farming is associated with increased *An*. *arabiensis* transmitted malaria incidence, because large adult females with high teneral reserves and decreased susceptibility to insecticide intoxication may also prove to be more efficient malaria vectors. In general, larval nutrient deprivation in *An*. *arabiensis* has important implications for subsequent adults in terms of their size and relative insecticide susceptibility, which may in turn impact on their malaria vector capacity in areas where insecticide based control measures are in place.

## Background

The “silver spoon” hypothesis of larval nutrition in holometabolous insects suggests that well nourished larvae become healthier adults that are better capable of survival in variable environments [1]. Larval food restriction in mosquitoes exerts several effects. These include a longer developmental period, with larvae taking a longer time to pupate [2]. A prolonged larval stage is generally associated with an increased risk of mortality as a consequence of predation, breeding site instability and/or human interference [3]. Adults that eclose from larvae with low teneral reserves are smaller [4] and require more blood-meals to produce eggs [5].

The effect of larval starvation on mosquito-borne disease transmission is a subject of debate. It is postulated that larger mosquitoes live longer, increasing their disease transmission potential [6]. It is also reported that a restricted larval diet resulted in a modest but significant increase in the lifespan of the yellow fever vector *Aedes aegypti*[7]. In malaria vectors, smaller mosquitoes are postulated to have greater concentrations of free malaria parasites in their systems because they are less capable of melanising them, resulting in a higher transmission potential [8-10]. Furthermore, the multiple blood-meals required by smaller mosquitoes results in increased host contact and a greater risk of disease transmission [11].

*Anopheles arabiensis* is one of the dominant malaria vectors in Africa, particularly in Southern and Eastern Africa [12]. It is exophilic and not as anthropophagic as its sibling vector, *An*. *gambiae*. Although not as efficient a vector as *Anopheles funestus*, with which it is often found in sympatry, it is as efficient a vector as *Anopheles gambiae* S form [13]. As a consequence of exophily, it is a difficult vector to control by indoor residual spraying and the use of insecticide-treated bed nets [14].

*Anopheles arabiensis* is associated with the “paddies paradox”. This occurs in those instances where malaria transmission is reduced in irrigated areas compared to non-irrigated areas, likely because *An*. *arabiensis* tend to replace the more efficient vector *An*. *funestus* in irrigated areas [15]. For example, rice farming has been shown to exert a significant effect on malaria vector population dynamics whereby *An*. *arabiensis* are most abundant when rice crops are immature [16] and after fertilising [17]. Where *An*. *arabiensis* and *An*. *funestus* occur in sympatry, planned agriculture appears to lower the incidence of *An*. *funestus* borne malaria, but tends not to affect *An*. *arabiensis* borne malaria [18].

The most dramatic example of agriculture on the life history of *An*. *arabiensis* concerns the effect of maize farming. Maize is a ubiquitous African crop which flowers during rainy seasons. *Anopheles arabiensis* larvae are enhanced by the presence of maize pollen [19], so much so that it even makes up for the effect of larval crowding [20]. Larvae fed on maize pollen alone produce adults comparable in size to laboratory-reared adults fed on optimal diets [19]. It is postulated that increased malaria incidences in maize-growing districts are due to pollen abundance in areas close to where the vectors breed. The larvae readily ingest the pollen which promotes growth. Maize is commonly grown near dwellings allowing healthy adult mosquitoes to participate in malaria transmission [19].

The effect of larval nutrition on the expression of insecticide resistance in anophelines remains largely unexamined. It has previously been shown that adult *Musca domestica vicinia* flies raised on goats’ milk are more resistant to DDT than those raised on cow’s milk or other diets. *Aedes aegypti* larvae reared on a diet of milk are slightly more susceptible to insecticides than those raised on wheat flour [21]. From studies in mammals such as rats, it has been shown that micronutrients such as niacin, riboflavin, pantothenic acid and pyridoxine are required for Phase I reactions, specifically the cytochrome P450 system. Phase II conjugation reactions are also dependent on various nutritional requirements [22]. It is, therefore conceivable that metabolically-mediated insecticide resistance can be affected by generalized nutritional deprivation induced by starvation. The aim of this study was to examine the effect of generalized nutrient deprivation by starvation on the development of *An*. *arabiensis* larvae as well as on the expression of insecticide resistance in the resulting adults.

## Methods

### Mosquito strains

The SENN *An*. *arabiensis* strain was colonized from material collected in Sennar, Sudan, in 1980 and was used as the baseline for this study. A DDT resistant strain, called SENN-DDT, was intensively selected from SENN. Although selected for resistance to DDT, SENN-DDT also shows resistance to permethrin, deltamethrin and malathion. SENN-DDT is fixed for the L1014S *kdr* mutation [23]. This sodium channel mutation results in reduced neuronal sensitivity to DDT and pyrethroids, leading to DDT and pyrethroid cross resistance [24]. Both strains are maintained under standard laboratory conditions as described in Hunt *et al.*[25] in the Botha DeMeillon insectary facility at the National Institute for Communicable Diseases/NHLS in Johannesburg.

### Larval fasting regime

A fasting regime was established for the larvae of both strains. In the first cohort, larvae were provided with the equivalent of 1 mg of food (powdered dog food and yeast [25]) per larva per day (Daily cohort). Second cohort larvae were provided with a single aliquot of food on alternate days (2 Day cohort) and third cohort larvae were fed once every three days (3 Day cohort). Control larvae were provided with 1 mg of food per larva three times per day (Control cohort).

### Larval development rates

The larval fasting regimes described beforehand were performed and the development of the larvae monitored. For each larval fasting regime described above as well the control feeding regime fifty first instar larvae were placed in distilled water. The development rate of the larvae was quantified by recording the time to emergence of the first pupae as well as the time to emergence of 50% or more of the pupae. The larval fasting regimes were implemented for the SENN and SENN-DDT strains. Ten replicates were performed for each of the three fasting regimes for each strain as well as the controls.

For all subsequent experiments larvae were mass reared either under the control feeding regime or the 2 day fasting regime for comparisons between the two. Adults that accrued were used to determine adult susceptibility to DDT, adult size (wing length), *kdr* status and enzyme activity. The 2 day feeding regime was selected because it proved to be the regime most reduced that still allowed an appreciable number of adults to emerge for subsequent analysis. Adults from the 3 day feeding regime had a high level of mortality during eclosion and were therefore not suitable for further analysis.

### Adult susceptibility to DDT

Lethal time to induce either 50% or 75% mortality (LT50/LT75) by exposure to DDT was determined by bottle bioassay for each cohort by gender [26]. In brief, 1 mg/ml technical grade DDT (Sigma Aldrich, 31041) was prepared in acetone and used to coat the inside surfaces of 250 ml glass bottles at a concentration of 1 mg DDT per bottle. Three day old non-blood fed adults were exposed to DDT for varying lengths of time: 2.5, 5, 10, 15, 20, 25, 30 minutes for the base SENN strain, and 2, 4, 8 and 16 hours for the resistant SENN-DDT strain. Males and females were exposed separately with 20 adults being used per bottle for each time interval. For the SENN strain 50 replicates were performed per time interval and for the SENN DDT strain 25 replicates were performed per time interval. After each time interval, the adults were removed from the bottle, placed into cups and provided with a 10% sucrose solution. Mortality was scored 24 hours post exposure. Control exposures included the use of bottles treated with acetone only. An environmental control consisted of unexposed mosquitoes in cups. Data were log transformed to allow for the calculation of lethal time (LT) using linear regression. The LT75 was calculated for each cohort of the susceptible SENN strain as LT50 was too short a period to calculate accurately for this strain.

### Determination of adult size and *kdr* status of each strain

Three factors were examined in order to explain the difference in DDT resistance levels between control and fasted cohorts: vigour tolerance as approximated by adult size, *kdr* status and metabolic enzyme activity.

Wing length was used as an approximation of adult size [8] and was used to determine whether adults that succumbed to DDT exposure differed significantly in size from those that survived the same treatment. The sizes of SENN adults from the 2 day larval feeding regime cohort that survived 2.5 minutes exposure to DDT (collected 24 hours post exposure) were compared to those that proved susceptible following 2.5 mins DDT exposure (also collected 24 hours post exposure). This procedure was also performed on the SENN control cohort. The sizes of SENN-DDT adults from the 2 day larval feeding regime cohort that survived a 16 hour exposure to DDT (collected 24 hours post exposure) were compared to those that proved susceptible following a 16 hour DDT exposure (also collected 24 hours post exposure). As with the SENN strain, the procedure was also performed on the control cohort. Fifty individuals were measured for each strain treatment and gender. Both wings from each test mosquito were removed and placed on a glass slide. Wing length was determined using a calibrated micrometer at 200X magnification by measuring the distance from the distal end of the alula to the tip, excluding fringe scales [27]. Wing lengths of the exposure survivors were compared to those that had died. Differences in wing lengths were also analysed between samples from the different fasting regimes and gender.

SENN-DDT adults exposed to DDT for the longest time period (16 hours) were preserved, with the survivors and the dead preserved separately for *kdr* analysis. Similarly, the survivors and the dead of the shortest exposure (2.5 minutes) for the susceptible SENN strain were preserved. Thirty individuals were collected from each of these four phenotyped groups. DNA was extracted from each whole specimen using the method described by Collins *et al.*[28]. The presence of the L1014F (*kdr West*) and L1014S (*kdr East*) point mutations was analysed by hydrolysis probe analysis as described by Bass *et al.*[24].

### Detoxification enzyme activity in association with larval nutrition

Absolute detoxification enzyme activities for the two-day fasted and control SENN and SENN-DDT adults were determined. For each gender and strain 50 three day old non-bloodfed adult females and males were assayed. Their enzyme activities were compared to the standard susceptible *An*. *arabiensis* KGB strain. The adults were homogenised in distilled water and used in subsequent enzyme assays. Proteins were quantified using the Bradford assay, with Bovine Serum albumin as a standard curve [29]. Cytochrome P450 activity was measured as a function of haeme peroxidase activity [30]. The activity of α and β-esterases as well as general glutathione S-transferase (GST) activity was also determined according to established protocols [31,32].

### Data analysis

Exposure data were not corrected by Abbott’s formula as all control mortalities were below 5%. All statistical analysis was performed using Statistix 7. Comparisons between two samples were analysed using 2 sample t-tests. Comparisons between multiple samples were performed using one way Analysis of Variance (ANOVA) with the Tukey comparison of means being used as a post hoc test. Significance was set at 95% confidence.

## Results

### Larval development

**Figure 1:**
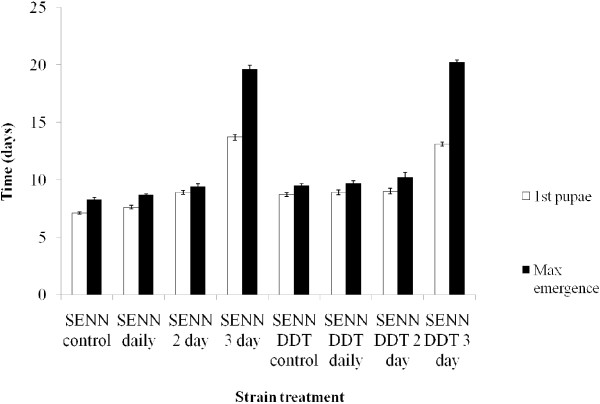
**Larval development times (expressed as days to first pupations and days to 50% pupation) by larval feeding cohort for the *Anopheles arabiensis *SENN and SENN**-**DDT laboratory colonies. **Cohort feeding regimes were thrice daily for the controls, once daily (SENN and SENN-DDT daily), once every second day (SENN and SENN-DDT 2^nd ^day) and once every third day (SENN and SENN-DDT 3^rd ^day). A total of 500 larvae per treatment were monitored, divided into 50 larvae per replicate.

Dietary restriction was generally associated with a decrease in the rate of larval development (Figure 1). The rate of larval development in the SENN cohorts fed once daily did not differ significantly from that of the control cohorts, but those SENN cohorts fed on alternate days and every third day took significantly longer for the first pupae to appear (ANOVA: p < 0.01, F = 235, df = 39). In SENN-DDT, however, only the cohort fed every third day developed significantly slower than the control (ANOVA: p < 0.01, F = 116; Tukey; Q = 3.81; df = 39). Comparing the control cohorts as well as cohorts fed daily between strains, SENN larvae developed significantly faster than SENN DDT larvae (ANOVA: p < 0.01, F = 19.7, df = 89). In the cohorts fed once either every two or three days, there was no significant difference in the rate of development between SENN and SENN-DDT.

### Adult susceptibility to DDT

**Figure 2:**
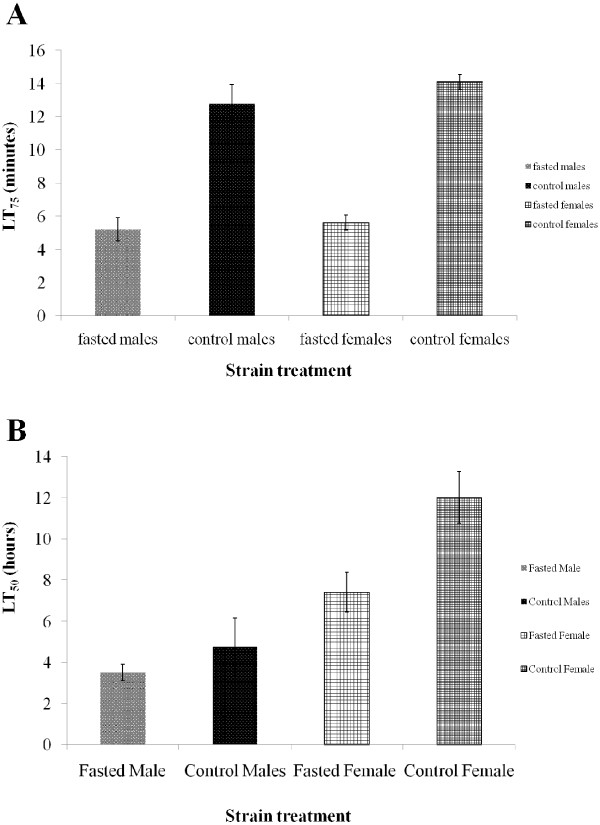
**(A) Mean lethal times (minutes) required to induce 75% mortality (LT75) in adult SENN *Anopheles arabiensis *by exposing them to DDT at a concentration of 1mg per 250 ml glass bottle. **Adults eclosed from fasted larvae (fed once every second day) are compared to adults eclosed from control larvae (fed thrice per day) by gender. (**B**) Mean lethal times (hours) required to induce 50% mortality (LT50) in adult SENN-DDT *Anopheles arabiensis *by exposing them to DDT at a concentration of 1mg per 250 ml glass bottle. Adults eclosed from fasted larvae (fed once every second day) are compared to adults eclosed from control larvae (fed thrice per day) by gender.

Adults that emerged from fasted larvae tended to have a reduced lethal time (LT) to 50% or 75% mortality during exposure to DDT compared to those from the controls. SENN fasted males showed a significantly reduced LT75 (2-sample t test: p < 0.01; t = 5.524), as did fasted females (p < 0.01; t = 13.51) (Figure 2A). This trend was partially mirrored in SENN-DDT (Figure 2B), where only fasted females showed a significantly reduced LT50 (p = 0.02, t = 3.54166). There was no significant difference in DDT susceptibility between SENN control males and females or between SENN fasted males and females. There was a significant difference in resistance to DDT between control SENN-DDT males and females (2-sample t test: p = 0.02; t = 3.542) as well as between SENN-DDT fasted males and females (2-sample t test: p = 0.02; t = 3.744).

### Adult sizes

**Figure 3:**
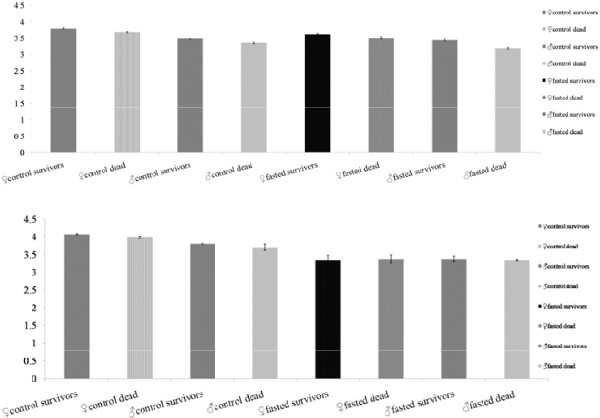
**(A) Wing lengths (mm) of *Anopheles arabiensis *SENN survivors and susceptibles (dead) following a 2.5 minute exposure to DDT. **Control cohorts (eclosed from larvae fed thrice daily) are compared to their corresponding fasted cohorts (eclosed from larvae fed once every second day) by gender. (**B**) Wing lengths (mm) of *Anopheles arabiensis *SENN-DDT survivors and susceptibles (dead) following a 16 hour exposure to DDT. Control cohorts (eclosed from larvae fed thrice daily) are compared to their corresponding fasted cohorts (eclosed from larvae fed once every second day) by gender.

**Figure 4:**
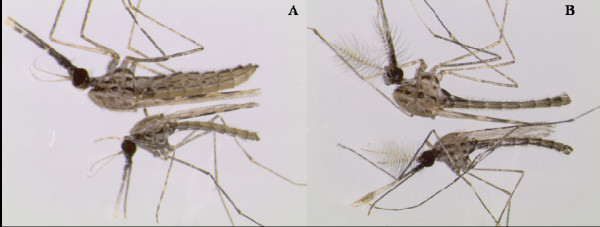
**Example of size variation between SENN *Anopheles arabiensis *adults eclosed from control larvae fed thrice per day (top) and those eclosed from larvae fed every second day(bottom).A** = females, **B** = males.

Control SENN survivors following exposure to DDT were significantly larger than those that succumbed by gender (ANOVA: p < 0.01; F = 40.2; df = 199) (Figure 3A). This was partially true for SENN-DDT, where only the female survivors were significantly larger than those that died (ANOVA: p < 0.01; F = 9.05, df = 199) (Figure 3B). Generally, fasted mosquitoes of either gender were smaller than their corresponding controls (Figures 3A and 3B) (ANOVA females: p < 0.01; F = 28.4; males: p < 0.01; F = 61.1; df = 199). Relative sizes of fasted adults in relation to their control counterparts are illustrated in Figure 4.

### *Kdr* status of each strain

The L1014S mutation was not detected in either the SENN or SENN-DDT strains. All SENN-DDT samples assayed for the L1014F mutation were homozygous resistant (L1014F/F), regardless of larval nutritional regime or whether they survived DDT exposure or not. All SENN adults were homozygous susceptible (wild-type L1014L/L), regardless of larval nutritional regime.

### Detoxification enzyme activity in association with larval nutrition

The mean absolute enzyme activities of each stain by feeding regime are given in Table 1. SENN control cohort enzyme activities of each enzyme class did not differ significantly from those of the corresponding KGB strain by gender, while the SENN-DDT control cohorts showed significantly higher enzyme activity levels for cytochrome P450 (ANOVA: p < 0.01, F = 19.1 for females; p < 0.01, F = 22.7 for males; df = 489), GST (ANOVA: p = 0.02, F = 4.28 for females; p < 0.01, F = 15.4 for males; df = 489), α-Esterase (ANOVA: p < 0.01, F = 8.79 for females; not upregulated in SENN-DDT males; df = 489) and β-esterase (ANOVA: p < 0.01, F = 9.89 for females; p < 0.01, F = 25 for males; df = 489). Female SENN-DDT controls generally showed the highest enzyme activity levels. When protein concentrations were standardised, fasted adults generally showed decreased rates of enzyme activity although not all enzyme classes were significantly decreased in all cohorts (Table 2). Fasted SENN males showed the greatest decrease in enzyme activity, with three out of four (Cytochrome P450, β esterase and GST) enzyme classes decreased. All other cohorts showed two out of four classes decreased. Cytochrome P450 and β esterase, both Phase I enzymes, were the most down-regulated in the fasted cohorts, with Glutathione S-transferases, Phase II enzymes, the least affected, only being significantly down-regulated in fasted SENN males.

**Table 1:** **Mean absolute detoxification enzyme activities for the SENN and SENN**-**DDT *An***. ***arabiensis *strains by larval feeding regime cohort by gender**

	**Cytochrome P450 equivalents/mg protein**	**α Esterase - α napthol equivalents/mg protein**	**β Esterase - β napthol equivalents/mg protein**	**Glutathione S-transferase**
				**μmol/min/ml**
	**(SD)**	**(SD)**	**(SD)**	**(SD)**
KGB♂	0.0046	0.0037	0.0044	0.000072
	(0.0012)	(0.0011)	(0.0008)	(0.00001)
KGB♀	0.0053	0.0048	0.0055	0.000103
	(0.0017)	(0.0013)	(0.0017)	(0.000041)
SBF♂	0.0047	0.0013	0.0049	0.000046
	(0.0018)	(0.0008)	(0.0016)	(0.00002)
SBF♀	0.0047	0.0017	0.0048	0.000048
	(0.0014)	(0.0007)	(0.0013)	(0.000017)
SBC♂	0.006	0.0017	0.0061	0.000057
	(0.0018)	(0.001)	(0.0021)	(0.00003)
SBC♀	0.0063	0.0039	0.0052	0.00007
	(0.0015)	(0.0017)	(0.0015)	(0.000072)
SDF♂	0.0105	0.0012	0.0038	0.000109
	(0.0034)	(0.0006)	(0.0006)	(0.0001)
SDF♀	0.0081	0.0041	0.0063	0.000121
	(0.0027)	(0.0023)	(0.003)	(0.000034)
SDC♂	0.0094	0.0014	0.0077	0.000139
	(0.003)	(0.0007)	(0.0014)	(0.000066)
SDC♀	0.0102	0.0066	0.0068	0.000144
	(0.0035)	(0.0034)	(0.0011)	(0.000041)

The KGB insecticide susceptible *An*. *arabiensis *strain was used as a baseline comparison against SENN fasted (SBF), SENN Control (SBC), SENN-DDT fasted (SDF) and SENN-DDT control (SDC). SD = standard deviation.

**Table 2:** **Comparison of enzyme activities of SENN and SENN**-**DDT *An***. ***arabiensis *adults eclosed from fasted larvae **(**2 day cohort**) **against their corresponding control cohorts **(**eclosed from larvae fed thrice daily**) **by gender**

	**SENN fasted ♂**	**SENN fasted ♀**	**SENN**-**DDT fasted ♂**	**SENN**-**DDT fasted ♀**
Cytochrome P450	↓(0.02)	↓(<0.01)	-	↓(0.02)
Alpha esterase	-	↓(<0.01)	↓(<0.01)	↓(<0.01)
Beta esterase	↓(<0.01)	-	↓(<0.01)	-
Glutathione-S tranferase	↓(0.02)	-	-	-

Statistical significance was assessed by 2 sample *t*-test. Significant decreases (↓) are accompanied by their respective p values in parentheses and hyphens indicate no statistically significant differences.

## Discussion

In general, food restriction at the larval stages of *An*. *arabiensis* slows the rate of larval development and affects body size and insecticide tolerance in adults. Similar effects of larval starvation on development rate and adult size have been recorded in the closely related *An*. *gambiae*[4].

There was some variation between *An*. *arabiensis* strains in terms of larval development rate. In particular, SENN control larvae developed faster than control larvae from the resistance selected SENN-DDT strain. This difference may be an inadvertent selection effect caused by intense selection for resistance to DDT in the SENN-DDT strain [33]. Nevertheless, these data show that the primary cause of a prolonged larval development rate in both strains was nutrient deprivation. This effect was most apparent in those larvae fed once every three days, slowing their developmental rate to nearly half that of the controls.

Larval nutrient deprivation significantly reduced subsequent tolerance to DDT in adult females from both strains as well as in SENN males. Surprisingly, the DDT tolerance of SENN-DDT adult males was not adversely affected following larval nutrient deprivation. This may be attributed to a general reduction in DDT tolerance in SENN-DDT males compared to females. The differences in tolerance to DDT between larval feeding regimes in SENN and SENN-DDT and between genders in SENN can be explained, at least in part, in terms of potential DDT resistance mechanisms and variation in adult body size.

SENN adults that died following DDT exposure were significantly smaller than the survivors. Furthermore, fasted adults were generally smaller than their corresponding control cohorts and males were generally smaller than females. Based on these data adult size is affected by gender and larval nutrition and is significantly associated with tolerance to DDT in SENN. These results were only partially mirrored in SENN-DDT where control female DDT survivors were larger than control female susceptibles, control females were generally larger than control males, and the control cohorts were larger than their corresponding fasted cohorts. There was no variation in adult size in association with DDT tolerance and gender in the fasted cohorts of SENN-DDT. This suggests that in the absence of a DDT resistance mechanism the effect of vigour tolerance, here expressed by adult size in which larger mosquitoes are more insecticide tolerant, is more pronounced.

DDT resistance in the selected SENN-DDT strain is likely based on a combination of L1014F *kdr* and enzyme mediated detoxification, although the relative contributions of each cannot be clearly quantified using the evidence currently available. The L1014F mutation has previously been described in *An*. *arabiensis* from Sudan [34] as well as in the SENN laboratory colony [23] although in both cases there was no clear correlation between L1014F genotype and DDT resistance phenotype. The L1014F mutation is fixed in SENN-DDT, confirming previously described data [35], and all detoxification enzymes classes were significantly up-regulated in SENN-DDT. However, larval nutrient deprivation was associated with significantly lower enzyme activities in adults from both strains, corresponding to reduced tolerance to DDT intoxication. This suggests that enzyme detoxification plays an important role in production of the DDT resistance phenotype in the SENN-DDT colony. Nevertheless, a link between DDT resistance and L1014F *kdr* in the SENN-DDT colony has been established based on the use of synergists [35] and is inferred by selection to fixation of L1014F using DDT.

The link between detoxification enzyme activity and the expression of DDT resistance/tolerance may be explained by examining the physiological effect of larval nutrient deprivation. When larval teneral reserves are depleted the larval fat body rearranges itself. There is a loss of mitochondria and rough endoplasmic reticulum, and there is a global reduction of protein as well as various endocrine disruptions [36]. As these effects are passed through to the adult stage, it is not surprising that adult enzyme activity levels in the starved cohorts are generally decreased, even after correction for total protein content. From the data presented here it is apparent that Phase I enzymes are the most affected, with cytochrome P450s and beta esterases showing the most significant reductions in activity. Interestingly, GST activity appears to be the least affected even though GSTs are primarily associated with resistance to DDT [37,38]. It is possible that the reduced Phase I detoxification enzyme activity in the fasted cohorts resulted in less detoxification by-product available for Phase II biotransformation, as opposed to the change in resistance in association with fasting being due to a direct reduction in Phase II GST-mediated detoxification.

This study was performed on laboratory strains, and a comparison of resistance profiles of specimens found in field conditions with varying access to larval food resources is needed to corroborate the results. Nevertheless, it is concluded that *An*. *arabiensis* larval development is prolonged by nutrient deprivation and that adults that eclose from starved larvae are smaller and less tolerant to DDT intoxication. This effect on DDT tolerance in adults is also associated with reduced detoxification enzyme activity most likely caused by larval nutrient deprivation. Conversely, well fed larvae develop comparatively quickly into large, more DDT tolerant adults. This could be important in those instances where cereal farming is associated with increased success of *An*. *arabiensis* as well as increased malaria incidence [19], because large adult females with high teneral reserves and decreased susceptibility to insecticide intoxication may also prove to be more efficient malaria vectors. In general, larval nutrient deprivation in *An*. *arabiensis* has important implications for subsequent adults in terms of their size and relative insecticide susceptibility, which may in turn impact on their malaria vector capacity in areas where insecticide based control measures are in place.

## Competing interests

The authors declare that they have no competing interests.

## Authors’ contributions

SVO assisted with the experimental design, conducted the experiments, analysed the data and produced the initial drafts of the manuscript. BDB assisted with the experimental design, analysis and interpretation of data and produced the final version of the manuscript. All authors read and approved the final manuscript.
